# Sustainable Remediation of Contaminated Soil Using Biosurfactants

**DOI:** 10.3389/fbioe.2021.635196

**Published:** 2021-03-15

**Authors:** Catherine N. Mulligan

**Affiliations:** Concordia Institute of Water, Energy and Sustainable Systems, Concordia University, Montreal, QC, Canada

**Keywords:** sustainable remediation, biosurfactants, indicators, organic contaminants, metal, metalloid

## Abstract

Selection of the most appropriate remediation technology must coincide with the environmental characteristics of the site. The risk to human health and the environment at the site must be reduced, and not be transferred to another site. Biosurfactants have the potential as remediation agents due to their biodegradability, low toxicity, and effectiveness. Selection of biosurfactants should be based on pollutant characteristics and properties, treatment capacity, costs, regulatory requirements, and time constraints. Moreover, understanding of the mechanisms of interaction between biosurfactants and contaminants can assist in selection of the appropriate biosurfactants for sustainable remediation. Enhanced sustainability of the remediation process by biosurfactants can be achieved through the use of renewable or waste substrates, *in situ* production of biosurfactants, and greener production and recovery processes for biosurfactants. Future research needs are identified.

## Introduction

Cost-effective solutions that require less resource are significant factors in determining the treatment of contaminated sites. Both *in situ* and *ex situ* treatment approaches are available, but the most appropriate selection must be sustainable and based on the site characteristics (Mulligan, 2019). The risk to human health and the environment must be reduced in all management steps. For example, for contaminated sediments, the risks of dredging, disposal, and/or potential beneficial reuse of the sediments must be determined. To work toward sustainability, indicators must be identified and quantified. During remediation, waste generation and landfill deposition must be minimized, natural resources conserved, and benthic habitats and wetlands protected (Yong et al., 2014). Landfills will continue to be filled with contaminated sediments and soil, and biodiversity in the environment will be reduced unless changes are made. Integrated innovative management practices need to be developed to ensure that the remediation practices are performed sustainably.

A potential solution for the pump and treatment method could involve the use of bio-based products such as biological surfactants instead of petroleum-based ones. Mulligan (2014b) has shown that biodegradable, non-toxic products called biosurfactants (e.g., rhamnolipids and sophorolipids) can be produced from waste materials and can be employed for soil flushing or washing for metal and organic contaminants or for enhanced biodegradation of organic pollutants. Biosurfactant applications for remediation of contaminated soil and water have potential based on low toxicity, high biodegradability, unlimited applicability, and relatively low production cost for sustainable remediation and critical micelle concentration (CMC) and high effectiveness in enhancing biodegradation and affinity for metals. Studies showed that for effective application of biosurfactants, they should be selected based on pollutant characteristics and properties, treatment capacity, costs, regulatory requirements, and time constraints. Moreover, understanding of the mechanisms of interaction between biosurfactants and contaminants can assist in selection of the appropriate biosurfactants for sustainable remediation. Enhanced sustainability of the remediation process by biosurfactants can be achieved through the use of renewable or waste substrates, *in situ* production of biosurfactants, and greener production processes of biosurfactants. Most research has involved rhamnolipids. Other biosurfactants and process scale-up need further investigation. Therefore, in this paper, the application of biosurfactants for remediation as a potentially more sustainable option and future research needs are discussed.

## Environmental Applications of Biosurfactants

### Rhamnolipids

Rhamnolipids are anionic due to their carboxylic moiety (Tan et al., 1994; Herman et al., 1995; Ochoa-Loza, 1998). Therefore, metals with positive charge can be removed by rhamnolipids added to soil and sediment as reviewed by Mulligan (2014b). Juwarkar et al. (2007) showed the rhamnolipid decreased toxicity and enhanced microbial activity (*Azotobacter* and *Rhizobium*) which showed improved soil quality, but cost effectiveness was not evaluated.

Rhamnolipid was evaluated for its ability to reduce soil ecotoxicology of an aged, contaminated soil (Slizovsky et al., 2011). By removing 39, 56, 68, and 43% of Zn, Cu, Pb, and Cd, the toxicity reduction of the treated soil was demonstrated by the increase in biomass levels and survival of two species of worms (*Eisenia fetida* and *Lumbricus terrestris*).

Although most studies have focused on cation removal, it has also been found that anions (chromium and arsenic) can also be removed. Massara et al. (2007) demonstrated the removal of Cr(III) by rhamnolipids from kaolinite and the reduction of Cr(VI) to Cr(III) within 24 days. Further research indicated hexavalent chromium extraction and reduction by rhamnolipids from contaminated water and soil (Ara and Mulligan, 2015). Mining residues were also studied for removal of the As(V) form, at high pH by rhamnolipids (Wang and Mulligan, 2009b). Cu, Zn, and Pb removal is also positively correlated with that of arsenic.

Another way to add a surfactant to contaminated soil is in the form of a foam. It could be potentially more efficient than a biosurfactant solution. A 0.5% rhamnolipid foam solution was evaluated for cadmium and nickel removal from a contaminated sandy soil (Mulligan and Wang, 2004) and for the treatment of fresh water sediments co-contaminated with polycyclic aromatic hydrocarbons (PAHs), Pb, Zn, and Ni (Alavi and Mulligan, 2011).

Congiu and Ortega-Calvo (2014) studied the influence of the rhamnolipid biosurfactant on PAH biodegradation. Two mechanisms for the biosurfactant use were identified: (a) micellar solubilization, which improved the PAH availability to microbial cells for biodegradation and (b) rhamnolipid partitioning into soil organic matter that increased the PAH desorption rate from the soil.

Mulligan and Roshtkhari (2016) showed that rhamnolipid and microbial cultures isolated from weathered oil could enhance flocculation of the oil sands tailings by a factor of 2.70. The mechanism of flocculation appeared to involve a hydrophobicity increase of the particles, followed by adsorption of the biosurfactants and other organic compounds to bridge between particles. The sedimentation of the tailings will allow reduction in the volume of the ponds.

A rhamnolipid-producing strain of *Lysinibacillus sphaericus* strain was studied by Gaur et al. (2019). The solubilities of various pesticides were enhanced including β endo-sulfan and ϒ hexachlorocyclohexane. The biosurfactant also had antimicrobial activities against six strains of pathogenic bacteria (*Aeromonas hydrophilia* MTCC 1143, *Bacillus subtilis* MTCC 441, *Escherichia coli* MTCC 723, *Klebsiella pneumonia* MTCC 109, *Pseudomonas aeruginosa* MTCC 424, and *Vibrio cholera* MTCC 3904).

### Surfactin

Surfactin is a lipopeptide produced by *B. subtilis* consisting of seven amino acids in a 14-carbon compound (Kakinuma et al., 1969). Surface tensions decreased to 27 mN/m with low surfactin concentrations (0.005%). Production costs are high due to low yields and expensive substrates. Various food by-products and wastes have been used as substrates including whey, sugar cane molasses, maize water, cashew apple juice, olive oil, and potato processing effluents (Mulligan, 2014a).

The two negative charges on the glutamate and the aspartate portions of surfactin enable the binding of various metals (Thimon et al., 1992). Subsequently, heavy metals desorption by surfactin from contaminated soil and sediments was demonstrated by batch washing experiments (Mulligan et al., 1999a). The mechanism of surfactin enhanced metal extraction was attachment to the soil interface and metal complexation by the biosurfactant, and subsequent detachment of the metal/micelle complex.

Other studies by Singh and Cameotra (2013) indicated that surfactin and a fungicide were produced by the strain *B. subtilis* A21. The lipopeptide was effective for the removal of both petroleum hydrocarbons (65%) and metals such as Cd, Co, Zn, Pb, Ni, and Cu (26–44%) under various conditions. Sorption on the soil of the biosurfactant decreased the efficiency by about 50%. Mustard seed germination after the soil washing process, was improved, indicating the environmentally friendly nature of the biosurfactant.

A study with two biosurfactants, surfactin and saponin, was performed to compare foam fractionation and soil washing for the removal of potential toxic metals (Cu, Zn, and Pb) from an industrial contaminated soil (Maity et al., 2013). Saponin and foam fractionation were more effective than surfactin and soil flushing. Pb was extracted more than Cu and Zn.

Das and Kumar (2018) studied an indigenous biosurfactant-producing *Bacillus licheniformis* strain for remediation of petroleum-contaminated soil. The biosurfactant was identified as a lipopeptide. Potato peel powder (an agroindustrial waste), in addition to the petroleum, was used to produce the biosurfactant. Experiments were carried out as a bioslurry. The toxicity reduction of the contaminated soil was determined through the earthworm toxicity test and the seed germination inhibition assay. The bioslurry treatment (500 g per 1 L water) with the microbial strain with and without potato peel powder successfully reduced the toxicity of the soil.

Isolation of a lipopeptide-producing strain of *B. subtilis* from a creosote-contaminated soil (Bezza and Chirwa, 2015) showed that the lipopeptide could recover 85% of the motor oil from sand. In biodegradation experiments, the lipopeptide enhanced the degradation of the oil by twofold. The lipopeptide was stable from pH 5 to 12, 25 to 125^*o*^C, and salinity of 5 to 20%. It also showed emulsification properties against of hexane and cyclohexane. Therefore, the lipopeptide has potential for enhanced oil recovery and petroleum-contaminated soil remediation.

Ashish (2018) studied the use of *Candida tropicalis* MTCC230 for its ability to enhance microbial enhanced oil recovery (MEOR) by a lipopeptide biosurfactant. The surface tension of water could be reduced to 32 mN/m. The CMC was 32.5 mg/L. The lipopeptide was stable under wide pH (2–12), temperature (30–90°C), and salinity (2–10%) ranges. Soil washing tests showed the ability to remove hydrocarbon contaminants from both water and soil. The suitability for MEOR was also indicated.

Felix et al. (2019) studied the use of cashew apple juice as a substrate for biosurfactant production for remediation of oil-contaminated soil. The biosurfactant lipopeptide reduced the surface tension of water and the interfacial tension with oil to 31.8 and 27.2 mN/m, respectively, with a CMC of 12.5 mg/L. The toxicity against lettuce and a microcrustacean was very low with a LC50 of 612 μg/mL. It was stable against pH, salinity, and temperature changes and was effective for remediation of oil-contaminated soil.

### Sophorolipids

The yeast *Candida bombicola* (formerly known as *Torulopsis bombicola*) produces a sophorolipid biosurfactant (Cooper and Paddock, 1984). The sophorolipid is produced in high yields which make it a potentially economic biosurfactant. Crude sophorolipids could potentially enhance metal removal from soils and sediments (Mulligan et al., 1999b, 2001).

Arab and Mulligan (2018, 2020) evaluated the use of sophorolipids for washing mining tailings. Increasing the temperature from 15 to 23°C increased removal of arsenic, copper, and iron, indicating its potential for remediation of mine tailings. In another study, Da Rocha et al. (2019) determined that the biosurfactant of *C. tropicalis* was much more effective for Zn and Cu removal than Pb. An economic analysis suggested the potential for industrial remediation by the biosurfactant. Ashish (2018) examined the application of a *C. tropicalis* biosurfactant-producing strain for remediation of motor oil contaminated sand.

Dispersion of biodiesel, diesel, and light crude-oil by sophorolipids was studied (Saborimanesh and Mulligan, 2018). Decreasing the surface and interfacial tension and micelle encapsulation of oil was determined as the main mechanism for the enhanced dispersion by the biosurfactant. Further study examined the biodegradability of these petroleum products by indigenous oil degrading bacteria with and without biodispersant addition (Saborimanesh and Mulligan, 2015). Characterization by 16S rRNA pyrosequencing indicated that *Firmicute* was the dominant phylum in the biodegradation of the biodiesel and diesel, whereas *Actinobacteria* in the diesel and *Proteobacteria* and *Actinobacteria in* the light crude oil. Addition of the sophorolipid enhanced the dispersion of the biodegradation of the hydrocarbons.

### Saponin and Other Biosurfactants

Plant-based non-ionic saponin is another biosurfactant that has been studied for removal of heavy metals from various soil types (Hong et al., 2002). Maximal cadmium and zinc removal from regesol was 90–100%, respectively. Unlike the previously discussed biosurfactants, saponins can be extracted from various plant parts such as the seeds, fruits, roots, and stems and are often classified as triterpenoids and steroid saponins. This wide distribution could make mass production easier and less costly (Kobayashi et al., 2012). For zinc, Mulligan et al. (2001) also found that saponin behaved in a similar manner to surfactin and rhamnolipid tests. Zeng et al. (2005) indicated that saponins can assist microorganisms for remediation by enhancing mass transfer and modifying cell hydrophobicity to enhance biodegradation. The surface tension of compounds like PAHs could be reduced.

Song et al. (2008) found that saponin was effective for removing phenanthrene and cadmium, from soil (87.7 and 76.2%, respectively). The mechanism for remediation of the organic contaminant, phenanthrene, was by solubilization and for cadmium, it was complexation with the carboxylic groups of saponin. At pH 6.5, saponin (2000 mg/L) was able to desorb 83% of the copper and 85% of the nickel from kaolin (Chen et al., 2008). Comparison to other agents showed the following: ethylenediaminetetraacetic acid (EDTA) > saponin > sodium dodecyl sulfate (SDS). The mechanism was adsorption of the surfactant, formation of metal ion pairs, and then desorption of the metal. More recently, Kobayashi et al. (2012) and Zhou et al. (2013) have shown that PAHs of three to five rings could be solubilized by saponin, and Cao et al. (2013) showed the desorption of PCB with Cu and Pb by saponin with ethylenediamine-N,N’-disuccinic acid (EDDS), while Ye et al. (2015) performed washing tests with a peanut oil-water solvent system with saponin for removal of polybrominated diphenyl ethers (PBDEs), polychlorinated biphenyls (PCBs), and PAHs and heavy metals from soil in conjunction with phytoremediation.

Liu et al. (2017) summarized the requirements for sustainable remediation with saponins. These included:

1. Model development for prediction of the ability of saponins to remove contaminants by biodegradation, flushing, or washing under a variety of conditions.
2. Improvement of purification and screening techniques for saponins.
3. Development of new applications regarding stabilization of nanoparticles for remediation.

A study by Rufino et al. (2013) showed that a yeast synthesized Rufisan biosurfactant which decreased the surface tension to 25.3 mN/m. Between 30 and 98% of the motor oil was removed from soil, respectively, by both the crude Rufisan biosurfactant and the purified biosurfactant at its CMC. The soil type and biosurfactant concentration did not affect the oil removal rate. Thus, the main mechanism of oil removal was likely oil displacement.

Resende et al. (2017) isolated a bacterial strain from seawater. The surface tension was reduced to 29 mN/m, and a maximum concentration of 3.6 g/L was produced. The strain was also very stable. A frying oil showed the best results for biosurfactant production. Motor oil could be removed by up to 90% from soil. Therefore, it has potential for future remediation.

Suryanti et al. (2016) examined the production of a glycolipid biosurfactant by *Rhodococcus rhodochrous* for remediation of cadmium. The biosurfactant had a CMC of 896 mg/L and could stabilize an emulsion up to 12 days. Both partially purified and crude biosurfactants were evaluated. The crude form could adsorb Cd slightly better than the purified form; thus, this form would be more economic.

## Discussion and Future Research Directions

The concept of industrial ecology is to protect the environment and conserve resources (Mulligan, 2019). Principles include use of renewable resources and conservation of materials for industrial activities, efficient industrial production processes including reduction, recovery, recycling, and reuse of waste, and effective management of wastes and emissions. Biosurfactant production and their application for soil remediation should be viewed in this light.

**FIGURE 1:**
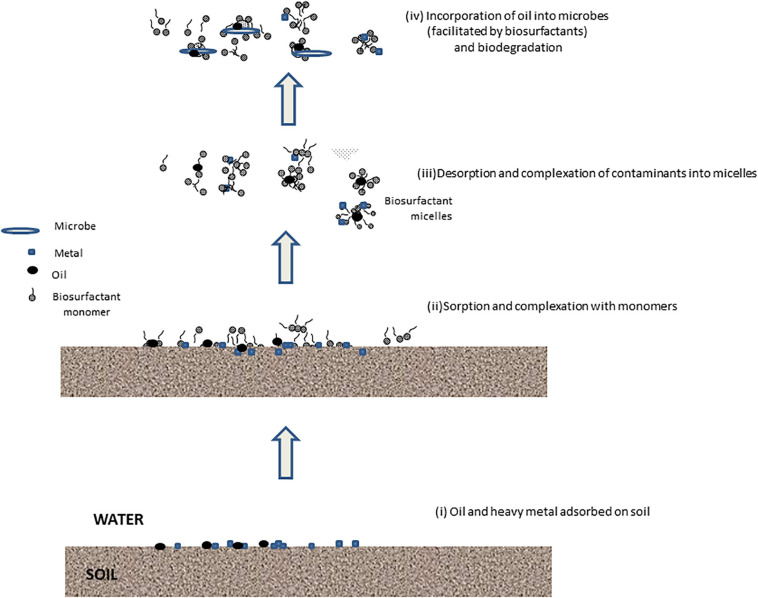
Interactions of biosurfactants with oil and metal contaminants on soil.

Various applications of biosurfactants for treatment of contaminated soils, sediment, and waste (e.g., tannery sludge and mining wastes) have been discussed. Some of the applications of biosurfactants for biodegradation for mixed contaminants are shown in Table 1A and for washing or flushing in Table 1B. By solubilization and emulsification of the contaminants, biosurfactants can enhance biodegradation of contaminants. Since the biosurfactants are biodegradable, biosurfactants remaining after treatment will not contribute toxicity to the treated soil. More research of more complex situations is needed, particularly regarding mixed organic and inorganic contamination. The mechanism of removal by the biosurfactants of oil and metal contamination is shown in Figure 1.

**TABLE 1:** Selected biodegradation studies and soil washing/flushing studies involving biosurfactants.

(A) Biodegradation studies					

Biosurfactant	Medium	Microorganism		Contaminant	References
Crude biosurfactant	Soil	*Bacillus subtilis* ICA 56		Hydrocarbons and heavy metals	Lima de Franca et al. (2015)
Rhamnolipid	Soil	*Luteibacter* sp.		Petroleum and heavy metals	Zhang et al. (2011)
Rhamnolipid	Soil	*P. chrysosporium*		PAHs	Wang et al. (2014)
Rhamnolipid	Sand	Indigenous microorganisms		PAHs, n-alkanes	Nikolopoulaou et al. (2013)
Rhamnolipid	Soil	*P. aeruginosa* DSVP20		Eicosane, fluoranthene, pristane	Sharma et al. (2015)
Rhamnolipid	Soil	Indigenous microorganisms		Diesel oil	Whang et al. (2008)
Rhamnolipid	Soil	*P. putida* ATCC 17484		Phenanthrene	Gottfried et al. (2010)
Rhamnolipid	Soil	Pyrene degrading bacteria		Pyrene	Jorfi et al. (2014)
Rhamnolipid	Soil	Indigenous microorganisms		Chloropyrifos	Singh et al. (2016)
Rhamnolipid	Sediment	Indigenous microorganisms		Triclosan	Qian et al. (2016)
Rhamnolipid	Soil	*P. chrysosporium*		Organochlorine pesticides	Wang et al. (2014)
Rhamnolipid	Soil	*Pseudomonas aeruginosa* A11		Hg, Ni	Singh and Cameotra (2013)
Lipopeptide	Soil	*Staphylococcus* sp.		High salinity crude oil	Hentati et al. (2021)
Saponin	Soil	*Burkholderia cepacia* RPH1		Phenanthrene	Choi et al. (2009)
Biosurfactants	Soil	*Enterobacteriae*, *Pseudomonas*, and other isolates		PAHs	Cazals et al. (2020)

**(B) Soil washing/flushing studies**					

**Biosurfactant**		**Medium**	**Contaminant**		**References**

Crude glycolipid		Soil	Cd		Suryanti et al. (2016)
Lipopeptide		Soil	Oil		Felix et al. (2019)
Lipopeptide		Sand	Motor oil		Bezza and Chirwa (2015)
Lipopeptide		Soil	Petroleum		Das and Kumar (2018)
Rhamnolipid		Sepiolite, feldspar	Cd		Aşçi et al. (2008a)
Rhamnolipid		Feldspar	Zn		Aşçi et al. (2008b)
Rhamnolipid, surfactin		Kaolinite	Pb		Kim and Vipulanandan (2006)
Rhamnolipid, MEL, saponin		Soil, sediment	Zn, Cu, Pb, Oil		Mulligan et al. (2007)
Rhamnolipid		Soil, water	Cr		Ara and Mulligan (2015)
Rhamnolipid		Mining residues	As		Wang and Mulligan (2009a, b)
Rhamnolipid		Sediments	PAH, Pb, Zn, Ni		Alavi and Mulligan (2011)
Rhamnolipid		Soil	Pesticides		Gaur et al. (2019)
Rhamnolipid, viscosin		Soil	PAHs, metals		Amani (2015)
Rhamnolipid		Soil and mining residues	Cr, Cu, and Ni		Barajas-Aceves et al. (2015)
with phytoremediation			
Rhamnolipid, citric acid		Garden soils	Cd, Pb, lindane		Wan et al. (2015)
Rhamnolipid		Mined soil	Fe		Akintunde et al. (2015)
Rhamnolipid		Sediments	Cd, Cr, Cr, Pb		Chen et al. (2017)
Rhamnolipid foam		Sandy soil	Petroleum, diesel oil		Da Rosa et al. (2015)
Rhamnolipid		Clay loam or sand	Oil		Hallmann and Medrzycka (2015)
Rhamnolipid+surfactin with		Soil	Oil		Fanaei et al. (2020)
H_2_O_2_ assisted biotreatment			
Rufisan		Soil	Motor oil		Rufino et al. (2013)
Saponin		Soil	PCB, Cu, Pb		Cao et al. (2013)
Saponin, tannic acid		Soil	As		Gusiatin (2014)
Saponin with phytoremediation		Soil	PBDEs, PCBs, PAHs,		Ye et al. (2015)
			Heavy metals		
Surfactin		Tannery sludge	Cr		Kilic et al. (2011)
Sophorolipids		Mining residues	As		Arab and Mulligan (2020)
Surfactin		Soil	Oil		Ashish (2018)
Surfactin, saponin		Soil	Cu, Zn, Pb		Maity et al. (2013)
Surfactin, fengycin		Soil	Cd, Co, Zn, Pb, Ni, Cu, petroleum hydrocarbon		Taira et al. (2015)

The high cost of producing the biosurfactants has limited full scale applications. This is due to low yields, rates of production, and recoveries (Descaro et al., 2017). The chemical properties of different congeners are also difficult to control. Genetic manipulation is underway to produce congeners appropriate for specific applications. Crude preparations and inexpensive or waste substrates can be employed (Mulligan and Gibbs, 1993). Waste materials as substrates will also improve the sustainability of the production process through cost reduction and waste reduction. As indicated by Marchant (2019), a full life cycle analysis (LCA) is necessary to identify where costs such as energy requirements during the fermentation process can be reduced. For example, Mulligan and Gibbs (1993) indicated that low-cost raw materials, increasing yield and production rates, optimization of the fermentor operation, reduction of product recovery costs, and matching the appropriate biosurfactant grade with the application will optimize the process costs such as employing crude instead of purified biosurfactants for environmental applications.

Biosurfactants thus can be produced externally and added to soil *in situ* or through soil washing. Subsequent recovery of the biosurfactants for reuse can enhance process sustainability. Liu et al. (2018) reviewed the substrates used by rhamnolipid producers. These include a variety of soluble sugars (glucose and glycerol), hydrocarbons (crude oil and diesel), and vegetable oils (e.g., coconut, palm, olive, etc.). For more sustainable production, waste substrates have been studied to reduce disposal issues and costs but can be inconsistent in quality. Some of these include water-mixable waste, molasses, whey milk or distillery waste peels of various fruits and vegetables, wastes from coffee and tea, whey, and waste cooking oils (Mulligan, 2014b).

Another approach is to biostimulate the microorganisms to produce the biosurfactants *in situ*. This reduces soil transportation costs and reduces risk of contaminant exposure and degrades the organic contaminants. Enhanced sustainability of the remediation process by biosurfactants can be achieved through the use of renewable or waste substrates, *in situ* production of biosurfactants, and greener production processes of biosurfactants. *In situ* biosurfactant production could be sustainable and cost effective due to the lower labor, material, energy, and transport requirements. Various biosurfactant-producing strains of *Bacillus* and *Pseudomonas* have been determined at hydrocarbon-contaminated sites (Jennings and Tanner, 2000) and thus stimulating *in situ* production could be strategic (Jalali and Mulligan, 2008). The role of *in situ* biosurfactant production could also enhance natural attenuation processes in the soil and groundwater (Yong and Mulligan, 2019). However, the understanding of the fate and transport of the contaminants with the biosurfactants in the subsurface studies needs to be improved. Injection of genetically modified organisms in an *in situ* application will likely not be acceptable both to regulatory authorities and to the public.

## Conclusion

In summary, biosurfactants have the potential for sustainable remediation of contaminated soils, sediments, and wastes (e.g., tannery and mining) due to their low toxicity, biodegradability, and effectiveness. However, the entire life cycle of the biosurfactant needs to be evaluated in order to optimize material, energy, and cost requirements. *In situ* production of the biosurfactants is potentially the most sustainable approach. Scale-up studies of the remediation process through partnership research and development are highly desirable.

## Author Contributions

The sole author CM prepared and edited the article completely with no assistance from anyone else.

## Conflict of Interest

The author declares that the research was conducted in the absence of any commercial or financial relationships that could be construed as a potential conflict of interest.
